# Novel programs, international adoptions, or contextual adaptations? Meta-analytical results from German and Swedish intervention research

**DOI:** 10.1186/1472-6963-14-S2-O32

**Published:** 2014-07-07

**Authors:** Henna Hasson, Knut Sundell, Andreas Beelmann, Ulrica von Thiele Schwarz

**Affiliations:** 1 Karolinska Institutet, Stockholm, Sweden; 2 Center for Epidemiology and Community Medicine, Stockholm, Sweden; 3 National Board of Health and Welfare, Stockholm, Sweden; 4 Friedrich-Schiller-University, Jena, Germany

## Background

One issue that remains to be solved in implementation research is adherence or adaptation dilemma [1,2]. In essence, this concerns to what degree and how evidence-based programs can be modified in accordance with restraints and possibilities in local context. This study compares effectiveness of program constructions (i.e., novel programs, adopted from other contexts with or without adaptations) in two meta-analytic data sets in two European countries.

## Materials and methods

Results are based on studies evaluating German child and youth preventative interventions (*n*=158), and Swedish psychological and social interventions (*n*=139). Interventions were categorized with three broad-band categories (novel programs, international adoption, and adaptation) and six sub-categories (innovation, conceptually new, adoption, cultural adaptation, pragmatic adaptation, and eclectic adaptation). All studies were coded by a trained coder followed by a second independent coding. The effect size of the outcomes of these program types were compared.

## Results

Novel programs, i.e. completely or conceptually new national programs, were the program type with the highest effect size in the German sample and among the highest in the Swedish sample. In both samples, international programs adopted without any adaptations were the least effective, even after controlling for crucial methodological aspects (design, sample size). Although adoptions proved to be effective (significantly different from zero), they were not as effective as the adapted or novel programs.

## Conclusions

The results favor novel and adapted programs and indicate that adoption of transported, international programs should not be done without considering adaptation. Adaptations justified explicitly for cultural reasons were more effective than international adopted programs without adaptations. This adds to the prior literature, which has shown contradictory results regarding the effects of cultural adaptations [3]. With respect to the high effect size for novel programs, it may be that a novel program encompasses the greatest possible fit to the context where it takes place. Novel programs may even involve tailoring the program to the needs of the specific setting, compared to internationally transported programs that are originally developed for another context.

## References

[B1] Stirman SW Miller CJ Toder K Calloway A Development of a framework and coding system for modifications and adaptations of evidence-based interventions Implementation Science 2013 14 65 10.1186/1748-5908-8-65 23758995 PMC3686699

[B2] Moore JE Bumbarger BK Cooper BR Examining adaptations of evidence-based programs in natural contexts The Journal of Primary Prevention 2013 14 147 161 10.1007/s10935-013-0303-6 23605294

[B3] Kumpfer KL Alvarado R Smith P Bellamy N Cultural sensitivity and adaptation in family-based prevention interventions Prevention Science 2002 14 241 246 10.1023/A:1019902902119 12387558

